# Membrane Docking Geometry of GRP1 PH Domain Bound to a Target Lipid Bilayer: An EPR Site-Directed Spin-Labeling and Relaxation Study

**DOI:** 10.1371/journal.pone.0033640

**Published:** 2012-03-30

**Authors:** Huai-Chun Chen, Brian P. Ziemba, Kyle E. Landgraf, John A. Corbin, Joseph J. Falke

**Affiliations:** Department of Chemistry and Biochemistry and the Molecular Biophysics Program, University of Colorado, Boulder, Colorado, United States of America; MRC National Institute for Medical Research, United Kingdom

## Abstract

The second messenger lipid PIP_3_ (phosphatidylinositol-3,4,5-trisphosphate) is generated by the lipid kinase PI3K (phosphoinositide-3-kinase) in the inner leaflet of the plasma membrane, where it regulates a broad array of cell processes by recruiting multiple signaling proteins containing PIP_3_-specific pleckstrin homology (PH) domains to the membrane surface. Despite the broad importance of PIP_3_-specific PH domains, the membrane docking geometry of a PH domain bound to its target PIP_3_ lipid on a bilayer surface has not yet been experimentally determined. The present study employs EPR site-directed spin labeling and relaxation methods to elucidate the membrane docking geometry of GRP1 PH domain bound to bilayer-embedded PIP_3_. The model target bilayer contains the neutral background lipid PC and both essential targeting lipids: (i) PIP_3_ target lipid that provides specificity and affinity, and (ii) PS facilitator lipid that enhances the PIP_3_ on-rate via an electrostatic search mechanism. The EPR approach measures membrane depth parameters for 18 function-retaining spin labels coupled to the PH domain, and for calibration spin labels coupled to phospholipids. The resulting depth parameters, together with the known high resolution structure of the co-complex between GRP1 PH domain and the PIP_3_ headgroup, provide sufficient constraints to define an optimized, self-consistent membrane docking geometry. In this optimized geometry the PH domain engulfs the PIP_3_ headgroup with minimal bilayer penetration, yielding the shallowest membrane position yet described for a lipid binding domain. This binding interaction displaces the PIP_3_ headgroup from its lowest energy position and orientation in the bilayer, but the headgroup remains within its energetically accessible depth and angular ranges. Finally, the optimized docking geometry explains previous biophysical findings including mutations observed to disrupt membrane binding, and the rapid lateral diffusion observed for PIP_3_-bound GRP1 PH domain on supported lipid bilayers.

## Introduction

In diverse cellular processes, a crucial step in pathway regulation is the generation of a signaling lipid within a specific membrane, which in turn recruits a wide array of signaling proteins to the surface of that membrane. The present study focuses on the second messenger lipid phosphoinositidyl-3,4,5-trisphosphate (PIP_3_), which is generated in the plasma membrane by the signaling enzyme phosphoinositide-3-kinase (PI3K) [1]–[14]. The array of proteins recruited to the plasma membrane by PIP_3_ are predominantly signaling proteins possessing PIP_3_-specific pleckstrin homology (PH) domains. Over 560 human proteins contain PH domains, many of which are lipid targeting domains that seek PIP_3_ or other PIP lipid variants on membrane surfaces [15]. A typical PIP_3_ signal recruits multiple PH domain-containing signaling proteins. In chemotaxis, for example, a PI3K-generated PIP_3_ signal at the leading edge of the plasma membrane recruits dozens of PH domain proteins involved in actin mesh regulation and membrane remodeling, thereby playing an essential role in driving the leading edge of the cell up an attractant gradient. More broadly, key cellular processes regulated by PIP_3_-triggered PH domain targeting include cell growth, DNA synthesis, cytoskeletal rearrangements, vesicle trafficking, and apoptosis [1]–[14], [16]–[19]. Mutations that alter this PIP_3_-specific membrane targeting are known to trigger disease, including cancer in humans [20], [21].

Despite the broad importance of PIP_3_-driven targeting of PH domains in cell signaling pathways, the membrane docking geometry of a PH domain bound to target PIP_3_ on a lipid bilayer has not yet been experimentally determined in any system. Previous studies have provided relevant structural insights, including: the crystal structures of dozens of co-complexes between a PIP_3_ headgroup analogue (inositol-1,3,4,5-tetraphosphate, IP_4_) and various PH domains [1], [22]; a solution NMR study of a PH domain bound to a short-chain PIP_3_ lipid embedded in a detergent micelle [23]; and a molecular dynamics study of a PH domain bound to PIP_3_ on a simple lipid bilayer [23]. Relevant biophysical information about protein-lipid interactions has also been provided by bulk equilibrium and stopped-flow kinetic studies of PH domains docking to target membranes [8], [24], [25], and by single molecule studies of the lateral diffusion of PIP_3_-associated PH domains in the membrane plane [26], [27]. Yet the currently available evidence is not sufficient to generate an accurate structural picture of the PH domain bound to its membrane-embedded target lipid, particularly with regard to the depth of the domain in the bilayer and its anglular orientation relative to the membrane plane. To address these structural questions it is necessary to experimentally determine the membrane docking geometry for a representative PH domain docked to a target bilayer containing PIP_3_.

**Figure 1:**
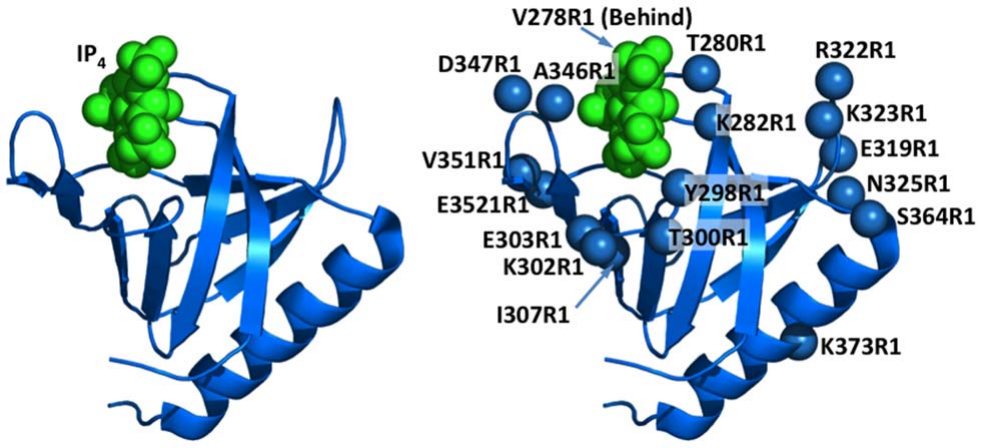
The GRP1 PH domain and positions chosen for spin labeling. (A) Domain topology, illustrating the secondary structure of GRP1 PH domain and the location of the PIP_3_ headgroup analogue (IP_4_) in the crystal structure of the co-complex (1FGY [22]). (B) The 18 sites selected for spin-labeling (blue spheres indicate Cα atoms), showing the high density of probe positions on the membrane docking face to provide optimal EPR analysis of the docking geometry. Figures generated in PyMol (DeLano Scientific LLC).

The present study focuses on the representative PIP_3_-specific PH domain of the General Receptor for Phosphoinositides 1 (GRP1, NCBI Gene ID 9265, CYTH3). GRP1 is an Arf6 guanidine-nucleotide exchange factor (GEF) that catalyzes the activation of Arf6-GDP to Arf6-GTP at the plasma membrane surface [28], [29]. The high resolution crystal structure of the co-complex between GRP1 PH domain and IP_4_ is known [1], [22]. The PH domain possesses a standard PIP_3_-specific headgroup binding pocket illustrated in Figure 1A and, adjacent to that pocket, a typical sentry glutamate excludes the constitutive plasma lipid phosphatidylinositol-4,5-bisphosphate (PIP_2_), enhancing the specificity for PIP_3_ [24], [25]. The domain core is a β-sandwich formed by two antiparallel β-sheets, and at one edge of the β-sandwich three inter-strand loops provide the basic side chains of the PIP_3_ binding pocket [1], [22], [30]. Additional basic side chains on the domain surface participate in an electrostatic search mechanism that senses the negative charge of the plasma membrane surface, provided mainly by phosphatidylserine (PS) lipids, to speed the rate of association with the rare PIP_3_ target lipid [8]. Once tightly bound to PIP_3_, the PH domain remains bound for seconds, and the diffusion of the protein-lipid complex in the membrane plane is remarkably rapid [26], [51]. The resulting lateral diffusion coefficient is indistinguishable from that of a single lipid molecule, indicating that the friction between the target lipid and the viscous bilayer (about 100-fold more viscous than H_2_O) is the limiting factor, while the protein interaction with the bilayer yields little additional friction. Such rapid diffusion of the PH domain likely speeds collisions between GRP1 and its membrane-bound effector proteins. While the GRP1 PH domain is currently the best studied representative, its structural and biophysical features appear to be shared by other important PIP_3_-specific PH domains, including AKT1 PH domain [24], [25].

To determine the membrane docking geometry of GRP1 PH domain bound to its target PIP_3_ lipid on a bilayer surface, the present study employs an established EPR approach involving site-directed spin labeling and spin relaxation measurements [31]–[42], [45]. The approach was derived from EPR studies measuring the membrane depths of lipid-exposed residues on transmembrane proteins [31]–[33], and has been adapted and successfully applied to multiple peripheral membrane binding proteins, including several Ca^2+^-regulated C2 domains [34]–[42]. For a peripheral protein bound to its target membrane, the approach measures the membrane penetration depths of a library of site-directed spin labels located at non-perturbing positions on the protein surface, then uses these constraints to position the protein in the bilayer, thereby defining both its penetration depth and angle relative to the membrane surface. The docking geometry provided by EPR analysis, in turn, can serve as an experimentally-defined starting point for subsequent molecular dynamics simulations designed to develop atomic resolution models of the membrane-docked protein [36], [43], [40], [46].

The present application determines the EPR membrane docking geometry of GRP1 PH domain bound to a simplified PC∶ PS∶ PIP_3_ target membrane containing both lipids essential for the native plasma membrane targeting reaction: (i) the target lipid PIP_3_ required for specific, high-affinity, equilibrium binding to the target membrane, and (ii) the facilitator lipid PS required for electrostatic searching and enhancement of the PIP_3_ on-rate [8]. The results reveal that the PH domain binds in a more shallow position than previously observed for C2 domains, made possible by the large size of the PIP_3_ headgroup that projects out from the membrane surface into solution. PH domain binding perturbs the position of the PIP_3_ headgroup away from its lowest energy, protein-free conformation, but the resulting headgroup conformation remains within the described range for PIP_3_ in bilayers [44]. Finally, the observed membrane docking geometry explains key features of GRP1 PH domain interactions with its target membrane observed in previous structural and biophysical studies.

## Results

### Strategy

In order to determine the EPR docking geometry of GRP1 PH domain bound to its target membrane surface, a fully functional Cysless variant (C293S/C327A/C343S) of human GRP1 PH domain that exhibits wild-type affinity for PIP3-containing target membranes [26] was employed as a background in which to create a suitable library of spin-labeled PH domains. Selection of spin-labeling positions was facilitated by the known structure of a co-complex between the PH domain and a soluble PIP_3_ headgroup analogue (IP_4_) [1], [22], which defined the general location of the membrane docking face. The 18 positions selected for Cys mutagenesis and spin label coupling were each solvent-exposed and did not contact the bound headgroup, thereby minimizing the risk of perturbed membrane binding. Most of the spin label positions (12/18) were targeted to the hemisphere containing the headgroup binding pocket, while the remaining control positions (6/18) were scattered across the other hemisphere. The functionalities of the resulting spin-labeled proteins were determined by measuring their relative affinities for target membrane. In previous studies such functional analyses have typically identified a small subset of spin labeled proteins that exhibit non-native membrane interactions [36], [40], justifying the exclusion of those proteins from subsequent EPR experiments.

The present EPR analysis began by measuring CW EPR spectra for each functional, spin-labeled PH domain in the free and membrane-bound states, in order to identify positions where membrane contacts trigger spectral changes. Subsequently, to directly determine the degree of membrane penetration, EPR depth parameters were measured for each functional, spin-labeled protein docked to target membrane and for calibration lipids in the same membrane background. These depth parameter measurements were designed to provide sufficient information, when combined with the known high-resolution protein structure, to generate a self-consistent membrane docking geometry that defines both the penetration depth and angle of the protein relative to the bilayer plane.

All biochemical and spectroscopic measurements described herein employed a physiological binding buffer (25 mM HEPES pH to 7.4 with KOH, 140 mM KCl, 15 mM NaCl, 0.5 mM MgCl_2_) and lipid bilayers containing both target PIP_3_ lipid and facilitating PS lipid in a simplified lipid mixture with the background lipid PC (FRET affinity titrations used PC∶ PS∶ PIP_3_∶ dPE in mole ratios 70∶ 23∶ 2∶ 5; EPR studies used PC∶ PS∶ PIP_3_ in mole ratios 74∶ 24∶ 2). The resulting model system provides a near-physiological membrane docking reaction, thereby maximizing the biological relevance of the EPR-defined membrane docking geometry.

### Site-Selection, Mutagenesis and Spin-Labeling of GRP1 PH Domain

The 18 positions selected for Cys incorporation and site-directed spin labeling in the fully functional Cysless GRP1 PH domain [26] are summarized in Figure 1B and Table 1. Each selected position is fully solvent exposed and lacks PIP_3_ headgroup contacts in the co-complex structure. The corresponding single-Cys mutations were introduced into the Cysless background by PCR site-directed mutagenesis, and PH domain was expressed and purified via its GST affinity tag. Spin labeling with methanethiolsulfonate spin-label (MTSSL), hereafter designated R1, was carried out while the protein was bound to the glutathione column, then the PH domain was washed and cleaved from the column by thrombin. The thrombin protease was affinity extracted and each concentrated, R1-labeled mutant was found to be 90 to 95% pure by SDS-PAGE.

**Table 1:** Site-directed spin label mutants of GRP1 PH domain and their measured parameters.

*PH Domain* 1	K_i_ ± SEM (µM)	*EPR Spectral Change* 2	Π (O_2_) ± SEM	Π (Ni) ± SEM	ϕ ± SEM
Wild-type	0.81±0.05	NA	NA	NA	NA
Cysless	0.97±0.20	NA	NA	NA	NA
V278R1 Lβ1-β2	1.20±0.08	++	0.25±0.01	0.17±0.02	0.31±0.02
T280R1 Lβ1-β2	0.70±0.16	++	0.27±0.05	0.53±0.03	−0.68±0.03
K282R1 β2	0.97±0.06	−	0.26±0.03	0.19±0.01	0.30±0.01
Y298R1 Lβ3-β4	1.37±0.05	+	0.38±0.02	1.18±1.11	−1.15±0.01
T300R1 Lβ3-β4	1.33±0.01	−	0.21±0.04	1.00±0.10	−1.57±0.01
K302R1 Lβ3-β4	0.63±0.16	+	0.22±0.01	1.12±0.04	−1.66±0.02
E303R1 Lβ3-β4	0.74±0.27	+	0.21±0.04	0.66±0.03	−1.19±0.01
I307R1 β4	1.01±0.28	+	0.17±0.01	0.77±0.09	−1.50±0.01
E319R1 Lβ5-β6	0.87±0.25	+	0.17±0.01	0.81±0.24	−1.52±0.01
R322R1 Lβ5-β6	1.69±0.09	++	0.31±0.03	0.29±0.04	0.10±0.02
K323R1 Lβ5-β6	0.93±0.29	+	0.22±0.06	0.78±0.06	−1.29±0.03
N325R1 β6	1.34±0.02	−	0.25±0.01	0.93±0.20	−1.30±0.03
A346R1 Lβi1-βi2	1.16±0.01	++	0.24±0.01	0.56±0.05	−0.87±0.01
D347R1 Lβi1-βi2	1.31±0.01	++	0.24±0.08	0.29±0.05	−0.23±0.01
V351R1 βi2	0.98±0.18	+	0.21±0.01	1.00±0.08	−1.58±0.02
E352R1 βi2	1.17±0.11	+	0.28±0.06	0.80±0.04	−1.09±0.03
S364R1 Lβ7-α1	1.02±0.09	−	0.22±0.04	1.35±0.03	−1.85±0.01
K373R1 α1	0.64±0.02	+	0.32±0.02	1.15±0.17	−1.29±0.01

1 For spin-labeled mutants (R1), the indicated residue in the Cysless PH domain is changed to Cys and labeled with MTSSL. Also indicated is the secondary structure element in which each spin label is located.

2 The qualitative ranking of spectral changes in Figure 4 utilizes three categories: large change (++), detectable change (+) and no detectable change (−).

### Effects of Spin Labels on Target Membrane Binding

**Figure 2:**
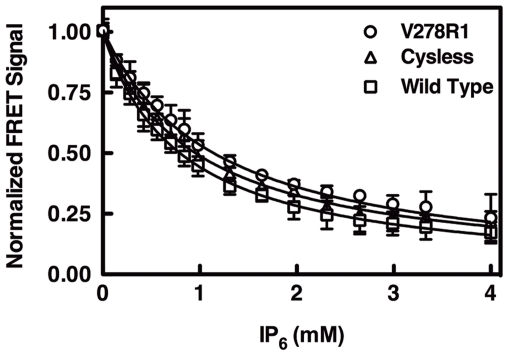
Effect of spin labeling on target membrane binding. Shown are representative competitive displacement curves for three GRP1 PH domains: Wild Type, Cysless and V278R1. Each PH domain was added to PC∶ PS∶ PIP_3_∶ dansylPE (mole ratios 70∶ 23∶ 2∶ 5) target membrane and allowed to form the PIP_3_-protein complex on the membrane surface. Subsequently, using a standard competition assay [8], [24], [25], the competitive inhibitor IP_6_ was titrated into the sample, thereby displacing PH domain from the membrane as revealed by decreasing protein-to-membrane FRET. The resulting competition curve was best fit for a homogeneous population of PIP_3_/IP_6_ binding sites (solid curves) to determine the K_i_ for IP_6_. Table 1 summarizes the measured K_i_(IP_6_) values, which are directly proportional to the affinity of each PH domain for membrane-embedded PIP_3_. Experimental conditions: 0.2 µM PH domain and 200 µM total lipid in 25 mM HEPES, 140 mM KCl, 15 mM NaCl, 0.5 mM MgCl_2_, pH 7.4, 25°C.

Previous studies have shown that site-directed spin labels introduced into membrane targeting domains are non-perturbing at most (>80%) surface positions not involved in specific lipid recognition sites [36], [40]. In order to identify any perturbing spin labels, the present study measured and compared the relative target membrane affinities of wild type, Cysless, and 18 spin-labeled PH domains in an established competitive displacement assay [8], [24], [25]. First, a given PH domain was added to target membranes containing PIP_3_ and PS to form the membrane-bound complex, then the competitive inhibitor IP_6_ (inositol-hexa-phosphate) was titrated into the sample to displace the PH domain from its target membrane. Displacement was monitored by an established protein-to-membrane FRET assay (Figure 2), yielding an equilibrium inhibition constant (K_i_) as summarized in Table 1. Notably, the K_i_ values of the wild type, Cysless and 18 spin-labeled PH domains differed by less than two-fold, indicating each of the surface-exposed, non-PIP_3_-coordinating spin labels had, at most, a minor effect (≤0.7 RT) on membrane docking. Thus, all 18 spin-labeled proteins were employed in subsequent EPR studies.

### Effects of Target Membrane Docking on EPR Spectra

**Figure 3:**
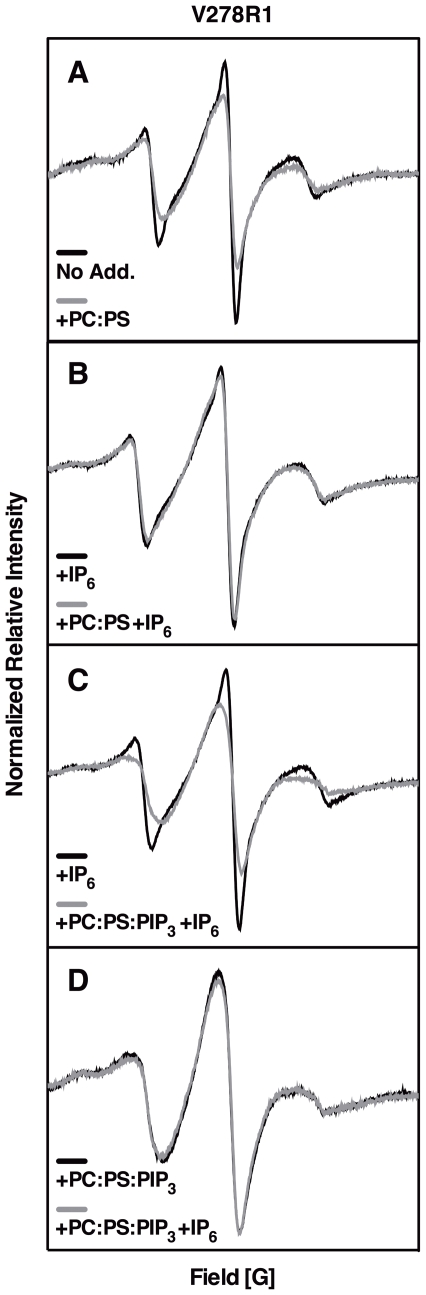
Control EPR spectra for a representative mutant. Shown are reproducible EPR spectral overlays for the MTSSL spin-labeled GRP1 PH domain V278R1, illustrating the strategy employed to analyze the spectral effects of membrane docking. (A) V278R1 PH domain in the absence and presence of control PC∶ PS (3∶1) membranes lacking PIP_3_, illustrating spectral broadening due to nonspecific membrane association. (B) V278R1 PH domain saturated with 200 µM IP_6_, both in the absence and presence of control PC∶ PS (3∶1) membranes, showing that unlike the apo PH domain the IP_6_-PH domain complex does not bind nonspecifically to membranes when PIP_3_ is absent. (C) V278R1 PH domain saturated with 200 µM IP_6_, both in the absence and presence of target PC∶ PS∶ PIP_3_ (74∶ 24∶ 2) membranes, showing the spectral change upon docking of the IP_6_-PH domain complex to membrane-bound PIP_3_ (with release of IP_6_). This is the standard comparison carried out for all spin-labeled PH domains (see Fig. 4), since the free IP_6_-PH domain complex does not dock to background lipids and use of this complex as a reference point ensures that spectral changes are due to the environmental effects of membrane docking, rather than to the conformational effects of ligand binding cleft occupancy. (D) V278R1 PH domain binding to target PC∶ PS∶ PIP_3_ (74∶ 24∶ 2) membranes in the absence and presence of saturating 200 µM IP_6_, showing that the competitive inhibitor IP_6_ does not perturb PH domain binding to target membrane PIP_3_ under these conditions. Each pair of overlayed spectra were obtained for two samples made from the same protein stock to ensure nearly identical spin concentrations, for which the same number of scans were collected and plotted in absolute intensity mode. Double integrations confirmed that each pair of spectra represented virtually identical numbers of spins. Thus, the relative intensities of each spectral pair can be directly compared. Spectra were acquired at 23°C and samples contained 10–200 µM protein, 0 or 40 mM total lipid as SUVs, and 0 or 200 µM IP_6_, in 25 mM HEPES, 140 mM KCl, 15 mM NaCl, 0.5 mM MgCl_2_, pH 7.4.

For each of the 18 functional spin-labeled PH domains, continuous-wave EPR spectra were collected and compared for (i) free domain in solution and (ii) domain in the presence of PC∶ PS∶ PIP_3_ target membranes. In both cases, the headgroup analogue IP_6_ was included at sufficient concentration to saturate the headgroup binding pocket when the PH domain was not bound to its preferred ligand PIP_3_. This approach prevented non-specific binding of the positively charged PH domain to the negatively charged membrane surface, since the large positive charge of the headgroup binding cleft was eliminated by the highly anionic IP_6_ ligand. In addition, the spectral changes observed upon addition of target membranes arose from membrane interactions rather than from a conformational change triggered by occupancy of the headgroup binding pocket, since the pocket was occupied in both its undocked and membrane-bound states (by IP_6_ in the free protein and by PIP_3_ in the membrane-bound protein). Figure 3 illustrates the importance of this strategy for a representative spin-labeled PH domain (V278R1, where R1 denotes the spin-labeled Cys side chain). The free PH domain binds detectably to PC∶ PS control membranes lacking the PIP_3_ target lipid, as indicated by the spectral broadening observed upon membrane addition (Fig. 3A). Inclusion of IP_6_ eliminates binding to control membranes (Fig. 3B) but has little or no effect on binding to PC∶ PS∶ PIP_3_ target membranes (Fig. 3D). In the latter experiment, the known 320-fold higher affinity of GRP1 PH domain for its target lipid PIP_3_ (K_D_ = 110±20 nm) compared to IP_6_ (K_D_ = 35±2 µM) [8] ensures the domain docks to its target lipid on the membrane surface even in the presence of IP_6_. Thus, the best way to detect spectral changes due to membrane docking is to compare the spectra of two samples that both contain a given PH domain and IP_6_, but either lack or contain target membrane, respectively (Fig. 3C).

**Figure 4:**
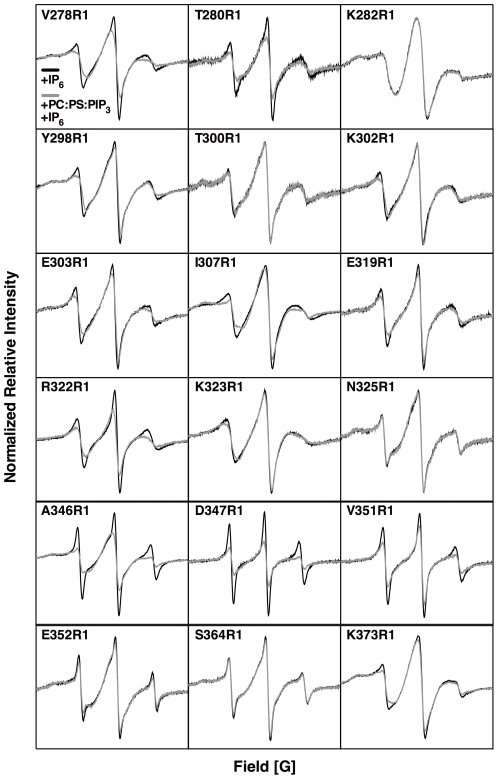
Effect of Target Membrane Docking on EPR Spectra. Each spectral overlay shows the effects of target membrane docking on the EPR spectrum of a given MTSSL spin-labeled GRP1 PH domain. The free PH domain was saturated with 200 µM IP_6_ and spectra were acquired in the absence and presence of target PC∶ PS∶ PIP_3_ (74∶ 24∶ 2) membranes. A spectral change is observed when the free IP_6_-PH domain complex docks to a target PIP_3_ headgroup on the membrane surface, releasing IP_6_. Since the ligand binding cleft is occupied in both states the spectral changes are triggered primarly by membrane docking rather than by cleft occupancy (see Figure 3). Table 1 qualitatively ranks the magnitudes of the target membrane-induced spectral changes (++, +, −). Each pair of overlayed spectra were collected as described in the Figure 3 legend thus their relative intensities can be directly compared. Spectra were acquired at 23°C and samples contained 10–200 µM protein, 0 or 40 mM total lipid as SUVs, and 200 µM IP_6_ in 25 mM HEPES, 140 mM KCl, 15 mM NaCl, 0.5 mM MgCl_2_, pH 7.4.

Figure 4 presents the EPR spectra of all 18 spin-labeled PH domains in samples containing IP_6_ and a) no membranes, or b) PC∶ PS∶ PIP_3_ target membranes. Table 1 summarizes the spectral changes when the free protein docks to target membranes. The 5 largest spectral changes, all broadenings, are observed for V278R1, T280R1, R322R1, A346R1, and D347R1, most likely arising from direct contacts between spin labels and target membrane (although indirect effects arising from membrane-triggered conformational changes cannot be ruled out). Smaller broadenings are detected at 9 other spin label positions, which could arise from subtler membrane contacts, or from docking-induced allosteric conformational changes, or from loss of rotational degrees of freedom when the freely tumbling PH domain docks to the membrane. No detectable spectral changes are observed at the remaining 4 positions, suggesting that spin labels at these positions remain fully solvent-exposed and/or retain extensive rotational mobility upon membrane docking, thereby preventing spectral perturbations due to altered environment or motions.

All EPR spectra were obtained using spin-labeled protein and target membrane concentrations that yielded virtually complete membrane docking of the protein population. Under these conditions, the membrane-bound proteins were separated by an average distance of ∼140 Å or more, thus spin-spin broadening (maximum range ∼20 Å under present conditions) was negligible.

### Measurement of Membrane Depth Parameters

To determine the membrane docking geometry of the PH domain bound to its PIP_3_ target lipid on the membrane surface, standard EPR power saturation methods were employed to measure the membrane depth parameters of both protein- and lipid-coupled spin labels in their target membrane-associated states [34]–[42]. Overall, membrane depth parameters were determined for a total of 22 spin-labeled molecules associated with PC∶ PS∶ PIP_3_ target membranes, including the 18 spin-labeled PH domains and 4 spin-labeled lipids, the latter used for depth calibration. The membrane depth parameter of a given spin label is defined by its relative accessibilities to a membrane-localized paramagnetic relaxation agent (O_2_) and an aqueous paramagnetic relaxing agent (the Ni^2+^ complex Ni^2+^EDDA^2−^). When the spin label collides with a paramagnetic agent, the EPR relaxation rate (1/T_1_) of the spin label is increased and the power required to saturate its EPR resonance increases correspondingly. Thus, to measure the depth parameter of a given, membrane-associated spin label, EPR power saturation was quantified for the spin label in otherwise identical membrane samples (i) containing ambient dissolved levels of O_2_, which preferentially partitions into the hydrophobic membrane interior, and (ii) purged with N_2_ to remove O_2_ but containing added Ni^2+^EDDA^2−^, which resides largely in aqueous regions. The resulting power saturation data yielded, for each spin label, an O_2_ accessibility parameter (Π(O_2_)) and a Ni^2+^EDDA^2−^ accessibility parameter (Π(NiEDDA)), that together defined the membrane depth parameter {Φ = ln [Π(O_2_)/Π(NiEDDA)]} (Methods, Eqn. 1). Highly positive depth parameter values indicate membrane burial with high O_2_ accessibility, while highly negative values indicate aqueous exposure with high Ni^2+^EDDA^2−^ accessibility [34]–[42].

Table 1 summarizes the measured accessibility and depth parameters. The six largest depth parameters observed for spin-labeled PH domains are V278R1 (Φ = 0.31±0.02), K282R1 (Φ = 0.30±0.01), R322R1 (Φ = 0.10±0.02), D347R1 (Φ = −0.23±0.01), T280R1 (Φ = −0.68±0.03), and A346R1 (Φ = −0.87±0.01). Notably, 5 of these 6 spin label positions also exhibit the 5 largest spectral shape changes observed upon target membrane docking (Fig. 4: V278R1, T280R1, R322R1 A346R1, and D347R1), consistent with a picture in which these 5 spin labels penetrate into the bilayer. The exception is K282R1 which displays a relatively large depth parameter (Φ = 0.30±0.01, Table 1) as would be expected for membrane penetration, yet the EPR spectrum of the free protein is quite broad even in the absence of membrane and does not change significantly upon membrane addition (Fig. 4). The simplest explanation is that K282R1 inserts into a protein cleft, both in the free and membrane-docked PH domain, such that its side chain tumbling is constrained and its nitroxide is protected from environmental changes and from Ni^2+^EDDA^2−^. The remaining 12 spin labels display solvent-exposed membrane depth parameters (Φ<−1.0, Table 1). Interestingly, the maximum depth parameters observed for the PH domain (Φ≤0.31) are significantly smaller than those observed for cPLA_2_ C2 domain (Φ≤2.4) and PKCα C2 domain (Φ≤1.3) measured under analogous conditions [36], [40], indicating the PH domain sits in a shallower position on the bilayer with considerably less protein penetration into the headgroup and hydrocarbon regions than typically observed for C2 domains.

### Modeling the Membrane Docking Geometry

In order to generate a structural picture of PH domain docked to the surface of the PC∶ PS∶ PIP_3_ target membrane, we employed a previously described procedure [36], [37], [40]. The measured depth parameters of the 4 spin labeled lipids provide depth calibration, since the depths of their spin labels in the bilayer have been experimentally determined. These calibration points, plus the known structure of GRP1 PH domain bound to its target PIP_3_ headgroup (IP_4_) and the modeled conformations of the 18 spin label side chains, are used to develop a self-consistent model that positions the protein crystal structure (1FGY [22]) in the membrane to optimize the agreement between the experimental depth parameters of individual spin labels and their modeled locations in the bilayer.

To prepare the known crystal structure coordinates of the GRP1 PH domain (1FGY [22]) for analysis of membrane docking geometry, cysteine residues with disulfide-linked, MTSSL spin label side chains were modeled at the 18 selected positions. Typically, the MTSSL side chain adopts a stable gauche^+^, gauche^+^ (g^+^, g^+^) conformation hydrogen bonded to the protein backbone, as observed in crystal structures [47]. Thus, the side chain conformation of each spin label was initially adjusted to this standard (g^+^, g^+^) configuration [47], which yielded sterically acceptable conformations for 16 of the 18 spin label positions. The remaining 2 positions (I307R1 and K323R1) exhibited steric clashes for the standard configuration, thus their geometry was further modified by rotations about the Cα-Cβ and Cβ-Sγ bonds of the side chains to minimize clashes (see Methods). The final conformations for I307R1 and K323R1 were (t, t) and (t, g^−^), respectively.

**Figure 5:**
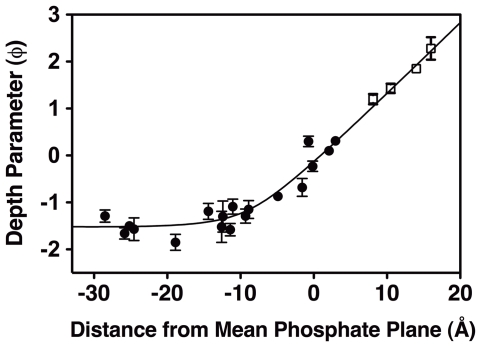
Hyperbolic relationship between spin label depth parameters and membrane penetration depths in the optimized, self-consistent EPR docking model. As described in Methods, the crystal structure of the GRP1 PH domain co-complex with IP_4_ (1FGY [22]) was modeled with MTSSL spin labels at the 18 chosen positions, then docked to the target bilayer using an interactive procedure that optimizes the known hyperbolic relationship between the measured spin label EPR depth parameters and the calculated spin label membrane penetration depths. Shown are the measured depth parameters for the protein spin labels (filled symbols) and the calibration lipid spin labels (open symbols), as well as the calculated membrane depth for each spin label in the final optimized, self-consistent EPR membrane docking model (Figure 6). The excellent agreement with the best-fit hyperbola (solid curve) emphasizes the high quality of the docking model. Depth parameters were measured by EPR power saturation (Methods) at 23°C and samples contained 10–200 µM protein, 40 mM total lipid as SUVs, 25 mM HEPES, 140 mM KCl, 15 mM NaCl, 0.5 mM MgCl_2_, pH 7.4. Except where otherwise indicated, errors are propagated from the errors of the accessibility parameters (Π(NiEDDA) and Π(O_2_)) used to calculate the depth parameter (Eq. 1), n≥15 power settings were used for each accessibility parameter measurement.

The self-consistent, best-fit membrane docking geometry was elucidated by iterative optimization using the available constraints. These included (i) the measured depth parameters and known penetration distances of the calibration spin-labeled lipids, (ii) the three-dimensional coordinates of the PH domain crystal structure modified as described above with the 18 spin-labeled side chains, and (iii) the measured depth parameters of these 18 spin probes. The model was based on an established hyperbolic relationship between the depth parameter and the distance from the center of the membrane bilayer [36], [37], [40]. Standard mathematical modeling software, Igor Pro (wavemetrics), was used to iteratively translate and tilt the PH domain structure relative to the membrane, while optimizing the fit of the protein and lipid constraints to the hyperbolic function. A small subset of 4 spin label positions (T280R1, E303R1, E352R1, S364R1) failed to yield good agreement with the best-fit hyperbola, suggesting their modeled side chain conformations were incorrect. Thus, during subsequent optimization, the conformations of these side chains were changed from (g^+^, g^+^) to a different, sterically acceptable comformation (T280R1 to (t, t); E303R1 to (g^+^, t); E352R1 to (g^+^, t); S364R1 to (t, t)) to improve the agreement with the best-fit hyperbola. Figure 5 presents the final, optimized distribution of measured depth parameters as a function of modeled membrane penetration distances, where each penetration distance is operationally defined as the distance from a given spin label nitrogen to the membrane plane representing the mean depth of phospholipid backbone phosphates [48]. Notably, the optimized data agree quite well with the best-fit, established hyperbolic relationship between the depth parameter and membrane penetration distance (Figure 5).

**Figure 6:**
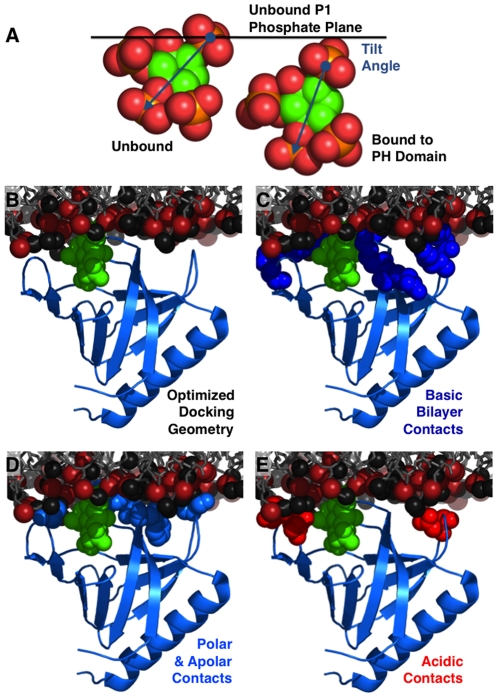
Protein-membrane interactions in the optimized, self-consistent EPR docking model. Shown is the optimized, self-consistent EPR docking model for GRP1 PH domain co-complexed with IP_4_ (1FGY [22]) and docked to a target bilayer. The schematic target bilayer highlights transient positions of backbone phosphates (red-brown spheres) and headgroups (PC or PS, black spheres) from a snapshot of a simulated bilayer [50]. (A) Views of the PIP_3_ headgroup relative to the mean backbone phosphate plane in both its lowest energy conformation (left) and its PH domain-bound conformation (right), illustrating the effect of PH domain binding on the target headgroup depth and orientation. (B) The PH domain docked to the schematic target bilayer in the optimized geometry. (C) Basic residues of the PH domain (dark blue spheres for R277, K279, K282, R283, R322, K323, R349) that can contact the negatively charged target bilayer in the optimized docking geometry. In some cases, the indicated side chain rotomer was adjusted to enhance membrane contact. (D) Hydrophobic and polar residues (light blue spheres for V278, T280, W281, P321, A346) that can contact the bilayer. Y298 obstructs the view and is not shown; it also contacts the bilayer and, perhaps more importantly, contacts multiple side chains responsible for specific PIP_3_ binding. (E) Acidic residues (red spheres for D320, E345, D347) that contact the anionic bilayer surface and are thus proposed to limit protein penetration into the target bilayer.

Figure 6 presents the optimized, self-consistent docking geometry of the target membrane-bound PH domain. The deepest protein backbone atom, Cα of residue V278, resides in the headgroup layer but is still shallower (by 2.4±2.6 Å) than the plane representing the average depths of headgroup backbone phosphates. The long axis of the core β-sandwich, operationally defined by the vector between the α-carbons of C292 and F296, lies at an angle 46±7° relative to the same plane. The backbone phosphate of the target lipid PIP_3_ headgroup bound to PH domain lies at a position 2.0±2.6 Å shallower than its normal depth [44] in the absence of PH domain, indicating the PH domain binding pulls the target lipid slightly towards the aqueous phase (Fig. 6A). PH domain binding also alters the angular orientation of the PIP_3_ headgroup, displacing the headgroup twist and tilt angles +17°±4° and +27°±4° relative to the optimal headgroup orientation [44], respectively, thereby tilting the headgroup towards the bilayer normal (Fig. 6A). Notably, however, both the depth and orientation of the PH domain-bound PIP_3_ are well within the energetically accessible range observed for free PIP_3_ in bilayers [44]. Thus, although PH domain binding displaces its target lipid away from its optimal configuration, the perturbation is small and not energetically costly. The transformations required to generate the optimized docking geometry from the crystal structure coordinates (1FGY [22]) are detailed in Methods.

### EPR Membrane Docking Geometry: Assumptions

The validity of the deduced EPR docking geometry relies on three assumptions, which are satisfied by the current study as follows. (I) The site-directed spin labels do not significantly perturb the protein-membrane interaction: this assumption is met since all 18 spin-labeled PH domains possess native-like affinities within 2-fold (or 0.7 RT) of wild type (see above). (II) The known backbone structure of the PH domain-IP_4_ co-complex does not change greatly upon membrane docking and (III) the modeled side chain conformations of the spin labels are reasonable: these assumptions are met since each modeled spin label side chain can be placed at or near a membrane depth consistent with its measured depth parameter (Fig. 5) without altering the backbone structure of the co-complex. Overall, the ability of the spin-labeled PH domains to satisfy the key assumptions is due likely to a combination of engineered and intrinsic features of the system: (a) each engineered spin label is carefully positioned to avoid PIP_3_ contacts, ensuring the integrity of the high affinity PIP_3_ binding pocket; (b) the structure of the membrane-associated PH domain bound to PIP_3_ co-complex is similar to that of the crystallographic co-complex between the PH domain and its target lipid headgroup, likely due both to the stable β-sandwich core of the PH domain and the multiple, strong coordination bonds between the domain and the tightly associated target headgroup, and (c) the preferred (g^+^, g^+^) geometry of the R1 side chain [47] is sufficient for self-consistency at 12 of the 18 library positions, while reasonable conformational variants suffice at the remaining 6 positions.

## Discussion

The membrane docking geometry determined by EPR for GRP1 PH domain bound to a PC∶ PS∶ PIP_3_ (74∶ 24∶ 2) target bilayer is well-defined, exhibiting best fit uncertainties of ±2.6 Å for the membrane penetration depth and ±1.9°, ±3.9° for the two PH domain rotation angles. The PH domain penetration into the bilayer is more shallow than previously observed for C2 domains: the deepest backbone Cα atom of the PH domain is V278 Cα, which resides in the headgroup zone but does not penetrate the plane of average lipid backbone phosphate positions (+2.4 ±2.6 Å from the average phosphate plane). By contrast, for representative lipid targeting C2 domains the deepest backbone Cα atoms do penetrate beyond the average phosphate plane, in each case inserting backbone into the glycerol backbone and hydrocarbon core zones of the bilayer (−7±1 Å for L37 Cα of cPLA_2_ C2; −5±3 Å for T250 Cα of PKCα C2; −5±3 for F234 Cα of SytI C2A; −3±3 for I367 Cα of SytI C2B) [36], [38]–[42].

The structural basis of the shallower PH domain penetration includes the geometry and chemistry of the target PIP_3_ headgroup, which is significantly larger and more negatively charged than the other headgroups of the plasma membrane inner leaflet, and thereby projects out significantly from the membrane surface into the aqueous phase [44]. As a result, the PIP_3_ headgroup can be engulfed by the PH domain with relatively little bilayer penetration. In the absence of bound protein, the PIP_3_ headgroup exhibits a lowest energy conformation defined by its equilibrium membrane depth and angular orientation [44]. GRP1 PH domain binding subtly translates the PIP_3_ headgroup toward solution (2.4±2.6 Å) and tilts the headgroup towards the bilayer normal (27°±4°), but this new headgroup conformation remains within the energetically accessible range [44]. It follows that the PIP_3_ headgroup conformation needed for PH domain binding would be well-sampled by normal headgroup motions, enabling rapid protein-headgroup association during a collision.

The EPR docking geometry sheds light on specific GRP1 PH domain residues that dominate the protein-membrane interactions, as summarized in Figure 6. The residues contacting the PIP_3_ headgroup (IP_4_) in the crystal structure of the PH domain-IP_4_ co-complex (1FGY [22]) are crucial to the nanomolar affinity binding of the PH domain to its target PIP_3_ headgroup: the resulting contacts anchor the conformations of 3 interstrand loops of the PH domain. Together with the stable β-sandwich core of the PH domain, these key loop constraints account for the ability of the crystallographic co-complex (Fig. 6B) to accurately describe the structure of the membrane-bound PH domain and to generate a self-consistent docking geometry exhibiting excellent agreement between the protein backbone and the depth parameters of the 18 spin label side chains in the PC∶ PS∶ PIP_3_ target bilayer (Fig. 5). In addition to the crystallographically-defined contacts between the PH domain and its target PIP_3_ headgroup, the EPR docking geometry in Figure 6C reveals that 7 basic side chains (R277, K279, K282, R283, R322, K323, R349) can contact the negatively charged bilayer surface, and thereby are ideally situated to assist with the electrostatic search mechanism that both speeds association with the rare PIP_3_ headgroup and enhances its nanomolar binding affinity [8]. Furthermore, 3 hydrophobic residues (V278, P321, A346) and 2 polar residues (T280, W281) contact the bilayer surface (Fig. 6D) and likely provide additional binding energy, particularly Trp281 that the docking model places within the bilayer region previously shown to yield stable indole binding [49]. Interestingly, the bilayer interaction appears to be limited by the negative charges of 3 acidic side chains (D320, E345, D347) that form a plane proximal to the bilayer surface, indicating these residues may have evolved, at least in part, to prevent deeper PH domain penetration into the negatively charged target membrane (Fig. 6E). Overall, the disposition of basic, acidic, hydrophobic and polar side chains relative to the bilayer makes good chemical sense, thereby corroborating the optimized EPR docking geometry.

Two additional lines of evidence from previous studies of GRP1 PH domain further support the EPR docking model. One line of evidence is provided by 3 mutations that weaken target membrane binding (V278E, Y298E, A346E) and by 1 mutation that has little or no effect on binding (V351E) [23]. The EPR docking model shows that the native V278, Y298, and A346 side chains contact the bilayer (in addition, Y298 appears to directly or indirectly stabilize three residues, K282, R284 and R305, that coordinate the PIP_3_ headgroup). By contrast, V351 does not contact the bilayer. Thus, the EPR docking model explains the effects of each mutation on binding. Furthermore, the strikingly shallow penetration of the EPR-docked PH domain into the bilayer is consistent with the remarkably rapid lateral diffusion observed in single molecule studies of PIP_3_-bound PH domain on PC∶ PS∶ PIP_3_ supported bilayers [51]. The PIP_3_-bound PH domain diffuses at a speed approaching that of a single lipid molecule, indicating that lipid interactions with the viscous bilayer dominate the diffusional friction, while protein interactions with the bilayer contribute little or no additional friction. By contrast, C2 domains that penetrate more deeply into the membrane exhibit significantly slower lateral diffusion than a single lipid due to their additional protein-bilayer interactions, which increase friction with the viscous bilayer (Ziemba, Knight & Falke, unpublished).

The EPR docking geometry model for GRP1 PH domain bound to a PC∶ PS∶ PIP_3_ target membrane provides molecular insights into the biological mechanisms and functions of the large class of PIP_3_-specific PH domains. In most cases, such PH domains share a conserved architecture and a homologous PIP_3_ binding cleft [1]–[14]. In addition, they are predicted to share an electrostatic search mechanism that enables the PH domain to more rapidly locate its rare PIP_3_ target lipid on the anionic plasma membrane surface [8], and are predicted to diffuse rapidly in the membrane plane once bound to their target PIP_3_ lipid [26], [51]. The present findings for GRP1 PH domain illustrate how a set of basic side chains can provide a positively charged protein surface for electrostatic searching, and how a PH domain can bind to a common conformer of the water-exposed PIP_3_ headgroup without penetrating deeply into the bilayer. The resulting rapid lateral diffusion of the PH domain-PIP_3_ complex is likely to be essential for fast reactions between membrane-associated PH domain-containing signaling proteins and their membrane-bound substrate lipids or effector proteins [26], [27]. In the case of GRP1 PH domain, the speed of membrane targeting and 2D diffusion ensure the GEF domain of the parent GRP1 molecule rapidly acquires its membrane-bound effector Arf6, ultimately yielding Arf6 activation. More broadly, rapid target acquisition and 2D diffusion is expected to be especially important for PH domain-containing proteins that, like GRP1, play central roles in fast signaling pathways such as chemotaxis.

## Materials and Methods

### Reagents

Synthetic 1-Palmitoyl-2-oleoyl-*sn*-glycero-3-phosphocholine (phosphatidylcholine, POPC, PC), synthetic 1-palmitoyl-2-oleoyl-*sn*-glycero-3-phosphoserine (phosphatidylserine, POPS, PS); and synthetic 1,2-dioleoyl-sn-glycero-3-phospho-(1′-myo-inositol-3′,4′,5′-trisphosphate) (DOPIP_3_) were all purchased from Avanti Polar Lipids; synthetic 1,2-dipalmitoyl-sn-glycero-3-phospho-(1′-myo-inositol-3′,4′,5′-trisphosphate) (DPPIP_3_) was purchased from Echelon. IP_6_ (inositol-1,2,3,4,5,6-hexaphosphate) were from Sigma. N-[5-(Dimethylamino)naphthalene-1-sulfonyl]-1,2-dihexadecanoyl-*sn*-glycero-3-phosphoethanolamine (dansyl-PE, dPE) was from Molecular Probes. 1-Oxyl-2,2,5,5-tetramethyl-Δ^3^-pyrroline-3-methylmethanethiosulfonate (MTSSL, R1) was from Toronto Research Chemicals. Ni^2+^-ethylenediamine diacetic acid (Ni^2+^EDDA^2−^, NiEDDA) was prepared as previously described [36], [40]. Spin label lipids 1-palmitoyl-2-stearoyl-(12-doxyl)-sn-glycero-3-phosphocholine (12 Doxyl PC), 1-palmitoyl-2-stearoyl-(10-doxyl)-sn-glycero-3-phosphocholine (10 Doxyl PC), 1-palmitoyl-2-stearoyl-(7-doxyl)-sn-glycero-3-phosphocholine (7 Doxyl PC) and 1-palmitoyl-2-stearoyl-(5-doxyl)-sn-glycero-3-phosphocholine (5 Doxyl PC) were from Avanti Polar Lipids.

### Protein Mutagenesis, Expression, Spin Labeling and Purification

The previously described optimized, fully functional Cysless GRP1 human PH domain (residues 255–392 and C293S/C327A/C343S) was employed as the background for creation of a single-Cys mutant library [26], [27]. Single cysteine mutants of were generated using the Quick Change II XL (Stratagene) site-directed mutagenesis kit according to the manufacturer's protocol. All mutations were verified by sequencing of the entire PH domain. The wild type, Cysless and mutant versions of the PH domain were expressed as GST-tagged fusions in *E. coli* as previously described [26], [27]. Protein was bound on a glutathione sepharose 4B resin (GE), washed extensively with a column wash buffer (150 mM Tris-HCl pH to 7.5 with HCl, 150 mM NaCl), followed by a wash step using the same buffer with NaCl increased to 0.5 M. When spin-labeled protein was desired, the protein-bound resin was further washed with reaction buffer (20 mM HEPES pH to 7.7 with KOH, 100 mM KCl) prior to labeling with 1 mM MTSSL for 60 min at 21°C via disulfide exchange. Following labeling, bound protein was washed and eluted off the column by cleavage of the GST-tag with thrombin (Novagen). Thrombin was affinity extracted from the protein sample using *p*-aminobenzamidine resin (Sigma).

### Preparation of Lipid Mixtures and Phospholipid Vesicles

All lipid components were mixed in solvent containing chloroform/methanol/water (1/2/0.8) to give the desired lipid ratios (below), dried under vacuum to remove all solvents, and then hydrated in assay buffer (25 mM N-(2-hydroxyethyl)piperazine-N′-2-ethanesulfonic acid (HEPES) at pH 7.4 with KOH, 140 mM KCl, 15 mM NaCl, and 0.5 mM MgCl_2_) by rapid vortexing. Small unilamellar vesicles (SUV) were generated by sonication of the hydrated lipid mixture to clarity with a Misonix XL2020 probe sonicator. Stock vesicles used in protein-to-membrane FRET assays were prepared with a total lipid concentration of 3 mM containing POPC∶ POPS∶ DOPIP_3_∶ dPE in the mole ratios 70∶ 23∶ 2∶ 5. Stock vesicles for EPR experiments were prepared with a total lipid concentration of 120 mM containing (i) POPC∶ POPS in the mole ratio 75∶ 25, or (ii) POPC∶ POPS∶ DPPIP_3_ in the mole ratios 74∶ 24∶ 2. Following sonication, vesicle stocks were allowed to equilibrate overnight at 4°C.

### Measurement of Relative Target Membrane Affinities by Titrations with a Competitive Inhibitor

A protein-to-membrane fluorescence resonance energy transfer (FRET) assay was used to measure the K_i_ for competitive displacement of PH domain from PIP_3_ on the target membrane as previously described [8], [24], [25]. The resulting K_i_ value is directly proportional to the affinity of the PH domain for its target membrane, enabling quantitative comparison of the relative target membrane affinities of wild type and modified PH domains. The assay makes use of the three intrinsic Trp residues of the PH domain as FRET donors, a dansylated lipid modified on its headgroup as FRET acceptor, and the soluble headgroup mimic inositol-1,2,3,4,5,6-hexaphosphate (IP_6_) as competitive inhibitor.

### EPR Spectra and Power Saturation Measurements

EPR spectra were acquired on a Bruker ELEXSYS E500 spectrometer (9.4 Ghz) equipped with a loop gap resonator (Molecular Specialties, Inc.) as previously described [36], [40]. Samples contained 10–200 µM spin-labeled PH domain along with the ligands and/or membranes indicated in the text.

EPR power saturation measurements to determine membrane depth parameters were carried out on the ELEXSYS E500 as previously described, yielding best-fit collision parameters for oxygen (Π(O_2_)) and Ni^2+^EDDA^2−^ (Π(NiEDDA)) [36], [40]. For a given spin label, both collision parameters were measured on the same day. Subsequently, these collision parameters were used to calculate the membrane depth parameter [31], [34], [36], [37], [40]:
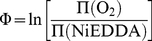
(1)where Φ is the measured depth parameter for a given spin labeled site.

### Determination of Membrane Docking Geometry Using the Measured Depth Parameters

A previously described modeling and iterative optimization procedure was employed to generate the EPR docking geometry for GRP1 PH domain bound to its target PIP_3_ lipid on the target bilayer [36], [37], [40]. Each of the 18 R1 side chains used for EPR depth parameter measurements were modeled (MacPyMOL, DeLano Scientific) in the PH domain crystal structure 1FGY as a Cys residue linked to the MTSSL spin label through a disulfide bond. All R1 sidechain conformations were initially adjusted to the standard dihedral angle of (g^+^, g^+^) or (+300°, +300°) about the first two side chain bonds, since this is the preferred R1 conformation in crystallographic studies of the T4 lysozyme [47]. Where appropriate, the R1 conformation was adjusted as described in the text.

The docking geometry and penetration depth of the PH domain was calculated by iterative fitting of the spin label depth parameters to an equation that models the dependence of the depth parameter on distance from the membrane center as a hyperbolic function [37]:
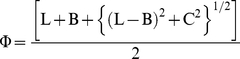
(2)where B represents the depth parameter value for a spin label distant from the center of the membrane and C defines the curvature of the function at the transition between linear and constant dependence of the depth parameter on distance. L describes the linear behavior of the depth parameter observed for spin labels in the membrane interior:
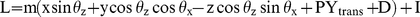
(3)where the m and I parameters represent the slope and intercept of the linear relationship. The x, y, and z coordinates tabulated from the nitoxide nitrogens of each spin label are represented by their respective letters and the associated θ_x_ and θ_z_ variables represent rotations about the x and z-axes of the coordinate system belonging to the nitroxide nitrogens. P is a Boolean variable set to unity for protein spin labels or zero for the calibration spin labels. D represents the known membrane depth of the spin-label, which is nonzero only for the calibration spin-labels. Altogether the equation used for fitting possesses one dependent variable (Φ), five independent variables (x, y, z, P and D) and seven unknown variables (θ_x_, θ_z_, Y_trans_, m, I, B and C). Nonlinear least-squares fitting of Eqn. 4 was performed using Igor Pro (Wavemetrics), yielding best-fit values of θ_x_, θ_z_, Y_trans_, m, I.

The algorithm used during the iterative fitting recognizes a laboratory axis system where the origin (0, 0, 0) is at the center-of-mass of the 1FGY structure and the x and y-axes are horizontal and vertical, respectively, in the viewing plane. An imaginary phosphate plane representing the average membrane depth of the phospholipid backbone phosphates is defined in this axis system as the x-z plane which passes through the origin (0, 0, 0), therefore the PH domain begins partially imbedded in the membrane interior. Subsequently, rotations about the x and z-axis, θ_x_ and θ_z_, along with a y-axis translation, are performed to optimize the docking angle and penetration with respect to the imaginary phosphate plane, which remains fixed during the transformations. Note that due to the planar symmetry of the membrane, translations along the x- or z- axis as well as rotations about the y-axis result in no change to the docking geometry of the models.

The optimized transformations, which yielded the final, self-consistent membrane docking geometry, can be carried out on the GRP1 PH domain crystal structure (1FGY [22]) to position the protein structure relative to the backbone phosphate plane. All rotations are performed from the perspective of an observer looking down the positive x or z-axis towards the origin. Specifically, the crystal structure was rotated counterclockwise −11.6 degrees (uncertainty ±1.9 degrees) about the x-axis, then counterclockwise −59.8 degrees (uncertainty ±3.9 degrees) about the z-axis, followed by a translation of −50.2 Å (uncertainty ±2.6 Å) along the y-axis to achieve a hyperbolic best-fit with a correlation coefficient R = 0.99.

## References

[1] Lemmon MA, Ferguson KM (2000). Signal-dependent membrane targeting by pleckstrin homology (PH) domains.. Biochem J.

[2] Czech MP (2000). PIP2 and PIP3: complex roles at the cell surface.. Cell.

[3] Insall RH, Weiner OD (2001). PIP3, PIP2, and cell movement–similar messages, different meanings?. Dev Cell.

[4] Vanhaesebroeck B, Leevers SJ, Ahmadi K, Timms J, Katso R (2001). Synthesis and function of 3-phosphorylated inositol lipids.. Annu Rev Biochem.

[5] Wang F, Herzmark P, Weiner OD, Srinivasan S, Servant G (2002). Lipid products of PI(3)Ks maintain persistent cell polarity and directed motility in neutrophils.. Nat Cell Biol.

[6] Czech MP (2003). Dynamics of phosphoinositides in membrane retrieval and insertion.. Annu Rev Physiol.

[7] DiNitto JP, Cronin TC, Lambright DG (2003). Membrane recognition and targeting by lipid-binding domains.. Sci STKE.

[8] Corbin JA, Dirkx RA, Falke JJ (2004). GRP1 pleckstrin homology domain: activation parameters and novel search mechanism for rare target lipid.. Biochemistry.

[9] Hennessy BT, Smith DL, Ram PT, Lu Y, Mills GB (2005). Exploiting the PI3K/AKT pathway for cancer drug discovery.. Nat Rev Drug Discov.

[10] Hurley JH (2006). Membrane binding domains.. Biochim Biophys Acta.

[11] Hawkins PT, Anderson KE, Davidson K, Stephens LR (2006). Signalling through Class I PI3Ks in mammalian cells.. Biochem Soc Trans.

[12] Newton AC (2009). Lipid activation of protein kinases.. J Lipid Res.

[13] Fayard E, Xue G, Parcellier A, Bozulic L, Hemmings BA (2010). Protein kinase B (PKB/Akt), a key mediator of the PI3K signaling pathway.. Curr Top Microbiol Immunol.

[14] Vadas O, Burke JE, Zhang X, Berndt A, Williams RL (2011). Structural basis for activation and inhibition of class I phosphoinositide 3-kinases.. Sci Signal.

[15] Finn RD, Mistry J, Tate J, Coggill P, Heger A (2010). The Pfam protein families database.. Nucleic Acids Res.

[16] Calleja V, Laguerre M, Larijani B (2009). 3-D structure and dynamics of protein kinase B-new mechanism for the allosteric regulation of an AGC kinase.. J Chem Biol.

[17] Calleja V, Alcor D, Laguerre M, Park J, Vojnovic B (2007). Intramolecular and intermolecular interactions of protein kinase B define its activation in vivo.. PLoS Biol.

[18] Okuzumi T, Fiedler D, Zhang C, Gray DC, Aizenstein B (2009). Inhibitor hijacking of Akt activation.. Nat Chem Biol.

[19] Wu WI, Voegtli WC, Sturgis HL, Dizon FP, Vigers GP (2010). Crystal structure of human AKT1 with an allosteric inhibitor reveals a new mode of kinase inhibition.. PLoS One.

[20] Carpten JD, Faber AL, Horn C, Donoho GP, Briggs SL (2007). A transforming mutation in the pleckstrin homology domain of AKT1 in cancer.. Nature.

[21] Lindhurst MJ, Sapp JC, Teer JK, Johnston JJ, Finn EM (2011). A mosaic activating mutation in AKT1 associated with the Proteus syndrome.. N Engl J Med.

[22] Lietzke SE, Bose S, Cronin T, Klarlund J, Chawla A (2000). Structural basis of 3-phosphoinositide recognition by pleckstrin homology domains.. Mol Cell.

[23] Lumb CN, He J, Xue Y, Stansfield PJ, Stahelin RV (2011). Biophysical and computational studies of membrane penetration by the GRP1 pleckstrin homology domain.. Structure.

[24] Landgraf KE, Pilling C, Falke JJ (2008). Molecular mechanism of an oncogenic mutation that alters membrane targeting: Glu17Lys modifies the PIP lipid specificity of the AKT1 PH domain.. Biochemistry.

[25] Pilling C, Landgraf KE, Falke JJ (2011). The GRP1 PH domain, like the AKT1 PH domain, possesses a sentry glutamate residue essential for specific targeting to plasma membrane PI(3,4,5)P(3).. Biochemistry.

[26] Knight JD, Falke JJ (2009). Single-molecule fluorescence studies of a PH domain: new insights into the membrane docking reaction.. Biophys J.

[27] Knight JD, Lerner MG, Marcano-Velazquez JG, Pastor RW, Falke JJ (2010). Single molecule diffusion of membrane-bound proteins: window into lipid contacts and bilayer dynamics.. Biophys J.

[28] Jackson TR, Kearns BG, Theibert AB (2000). Cytohesins and Centaurins: Mediators of PI 3-Kinase Regulated Arf Signaling.. Trends in Biochemical Science.

[29] Cohen LA, Honda A, Varnai P, Brown FD, Balla T (2007). Active Arf6 Recruits ARNO/Cytohesin GEFs to the PM by Binding Their PH Domains.. Molecular Biology of the Cell.

[30] Park WS, Heo WD, Whalen JH, O'Rourke NA, Bryan HM (2008). Comprehensive identification of PIP3-regulated PH domains from C. elegans to H. sapiens by model prediction and live imaging.. Mol Cell.

[31] Altenbach C, Greenhalgh DA, Khorana HG, Hubbell WL (1994). A collision gradient method to determine the immersion depth of nitroxides in lipid bilayers: application to spin-labeled mutants of bacteriorhodopsin.. Proc Natl Acad Sci U S A.

[32] Yu YG, Thorgeirsson TE, Shin YK (1994). Topology of an amphiphilic mitochondrial signal sequence in the membrane-inserted state: a spin labeling study.. Biochemistry.

[33] Ball A, Nielsen R, Gelb MH, Robinson BH (1999). Interfacial membrane docking of cytosolic phospholipase A2 C2 domain using electrostatic potential-modulated spin relaxation magnetic resonance.. Proc Natl Acad Sci U S A.

[34] Frazier AA, Wisner MA, Malmberg NJ, Victor KG, Fanucci GE (2002). Membrane orientation and position of the C2 domain from cPLA2 by site-directed spin labeling.. Biochemistry.

[35] Frazier AA, Roller CR, Havelka JJ, Hinderliter A, Cafiso DS (2003). Membrane-bound orientation and position of the synaptotagmin I C2A domain by site-directed spin labeling.. Biochemistry.

[36] Malmberg NJ, Van Buskirk DR, Falke JJ (2003). Membrane-docking loops of the cPLA2 C2 domain: detailed structural analysis of the protein-membrane interface via site-directed spin-labeling.. Biochemistry.

[37] Malmberg NJ, Falke JJ (2005). Use of EPR power saturation to analyze the membrane-docking geometries of peripheral proteins: applications to C2 domains.. Annu Rev Biophys Biomol Struct.

[38] Rufener E, Frazier AA, Wieser CM, Hinderliter A, Cafiso DS (2005). Membrane-bound orientation and position of the synaptotagmin C2B domain determined by site-directed spin labeling.. Biochemistry.

[39] Herrick DZ, Sterbling S, Rasch KA, Hinderliter A, Cafiso DS (2006). Position of synaptotagmin I at the membrane interface: cooperative interactions of tandem C2 domains.. Biochemistry.

[40] Landgraf KE, Malmberg NJ, Falke JJ (2008). Effect of PIP2 binding on the membrane docking geometry of PKC alpha C2 domain: an EPR site-directed spin-labeling and relaxation study.. Biochemistry.

[41] Kuo W, Herrick DZ, Ellena JF, Cafiso DS (2009). The calcium-dependent and calcium-independent membrane binding of synaptotagmin 1: two modes of C2B binding.. J Mol Biol.

[42] Kuo W, Herrick DZ, Cafiso DS (2011). Phosphatidylinositol 4,5-bisphosphate alters synaptotagmin 1 membrane docking and drives opposing bilayers closer together.. Biochemistry.

[43] Jaud S, Tobias DJ, Falke JJ, White SH (2007). Self-induced docking site of a deeply embedded peripheral membrane protein.. Biophys J.

[44] Li Z, Venable RM, Rogers LA, Murray D, Pastor RW (2009). Molecular dynamics simulations of PIP2 and PIP3 in lipid bilayers: determination of ring orientation, and the effects of surface roughness on a Poisson-Boltzmann description.. Biophys J.

[45] Frazier AA, Wisner MA, Malmberg NJ, Victor KG, Fanucci GE (2002). Membrane orientation and position of the C2 domain from cPLA2 by site-directed spin labeling.. Biochemistry.

[46] Lai CL, Landgraf KE, Voth GA, Falke JJ (2010). Membrane docking geometry and target lipid stoichiometry of PKCalpha C2 domain: A combined molecular dynamics and experimental study.. J Mol Biol.

[47] Langen R, Oh KJ, Cascio D, Hubbell WL (2000). Crystal structures of spin labeled T4 lysozyme mutants: implications for the interpretation of EPR spectra in terms of structure.. Biochemistry.

[48] Wiener MC, White SH (1992). Structure of a fluid dioleoylphosphatidylcholine bilayer determined by joint refinement of x-ray and neutron diffraction data. III. Complete structure.. Biophys J.

[49] Yau WM, Wimley WC, Gawrisch K, White SH (1998). The preference of tryptophan for membrane interfaces.. Biochemistry.

[50] Hoff B, Strandberg E, Ulrich AS, Tieleman DP, Posten C (2005). 2H-NMR study and molecular dynamics simulation of the location, alignment, and mobility of pyrene in POPC bilayers.. Biophys J.

[51] Ziemba BP, Knight JD, Falke JJ (2012). Assembly of membrane-bound protein complexes: Detection and analysis by single molecule diffusion.. Biochemistry.

